# Spatial access to residential care resources in Beijing, China

**DOI:** 10.1186/1476-072X-11-32

**Published:** 2012-08-09

**Authors:** Yang Cheng, Jiaoe Wang, Mark W Rosenberg

**Affiliations:** 1 School of Geography, Beijing Normal University, 19 Xinjiekouwai Avenue, Beijing, 100875, China; 2 Key Laboratory of Regional Sustainable Development Modeling, Institute of Geographic Sciences and Natural Resources Research, Chinese Academy of Sciences, 11A Datun Road, Chaoyang District, Beijing, 100101, China; 3 Department of Geography, Queen’s University, Kingston, ON, K7L 3N6, Canada

**Keywords:** Spatial accessibility, Residential care, Shortest path analysis, Two-step floating catchment area method, Planning

## Abstract

**Background:**

As the population is ageing rapidly in Beijing, the residential care sector is in a fast expansion process with the support of the municipal government. Understanding spatial accessibility to residential care resources by older people supports the need for rational allocation of care resources in future planning.

**Methods:**

Based on population data and data on residential care resources, this study uses two Geographic Information System (GIS) based methods – shortest path analysis and a two-step floating catchment area (2SFCA) method to analyse spatial accessibility to residential care resources.

**Results:**

Spatial accessibility varies as the methods and considered factors change. When only time distance is considered, residential care resources are more accessible in the central city than in suburban and exurban areas. If care resources are considered in addition to time distance, spatial accessibility is relatively poor in the central city compared to the northeast to southeast side of the suburban and exurban areas. The resources in the northwest to southwest side of the city are the least accessible, even though several hotspots of residential care resources are located in these areas.

**Conclusions:**

For policy making, it may require combining various methods for a comprehensive analysis. The methods used in this study provide tools for identifying underserved areas in order to improve equity in access to and efficiency in allocation of residential care resources in future planning.

## Background

Residential care refers to long-term care given to older population (aged 60 and over in this research) in a residential setting rather than at their homes. The settings include retirement homes, assisted living facilities, rest homes, and so on. Residential care as an alternative option for elder care has rapidly developed in Beijing in recent years. Demographic changes have been one of the major factors for the expansion of residential care services. The population aged 60 and over increased from 1.70 million in 2000 (12.5% of the total population) to 2.35 million in 2010 in Beijing (18.7% of the total population). Meanwhile, the oldest-old (aged 80 and over) increased from 132,928 in 2000 (0.98% of the total population) to 351,000 in 2010 (2.8% of the total population) [1,2]. The population aged 60 and over is expected to be 4.15 million (30% of the total population) in 2025 [3].

Increases in the older population are generating demand for a wide range of health and elder care services, including residential care. A second major factor generating demand is an official policy to support the development of residential care services. The Beijing Municipal Government aims to create a city – district (county) – subdistrict (township) multi-level residential care system and provide available residential care services for four per cent of the older population by 2020. The number of beds offered by residential care facilities (RCFs) almost doubled from 34,354 in 2006 to 67,018 in 2010 [1,4]. In order to meet the demand for four per cent of the older population, the number of beds will need to increase by 10,000 beds per year from 2010 to 2020 [5].

With the rapid growth of residential care resources, governments need to make greater efforts to ensure equal access to residential care services according to the geographic distribution of older people and the variety of demands they generate. From a geographic perspective, how to allocate residential care resources rationally and use knowledge-based planning for the future requires a better understanding of the distribution pattern of supply, demand, and spatial accessibility to resources by the older population. Availability of more and better data and the development of GIS technologies enable better measurement of spatial access to health care and social services and allows for the identification of underserved areas.

This study aims to measure spatial access to residential care resources in Beijing by using two GIS-based methods: shortest path analysis and a two-step floating catchment area (2SFCA) method. The specific research questions analysed are:

1) What are the distribution patterns of RCF service areas?

2) How do various factors affect spatial accessibility to residential care resources?

The second part of the paper systematically reviews the existing literature on the concept of access to health care and social services, geographic distribution and policy implications for residential care, and the application of GIS-based measurements of spatial access to health care and social services in English-speaking countries and China. In the third part, the data sources are described and the two methods are explained in detail that were used to measure service areas and spatial accessibility to residential care resources. The results section presents the travel times to RCF service, service areas of each RCF and the availability of residential care resources at the subdistrict (township) level, as well as spatial accessibility to residential care resources taking into account distance and size of RCFs. The paper ends with a discussion of the policy implications of the study methods and results. Limitations of the study are also addressed.

### Literature review

An extensive literature on access to health care services and location-allocation problems exists. With regard to the concept of access, researchers have defined access from various aspects. Rosenberg interpreted access as two components: economic and physical access [6]. The former is the ability to purchase health care services, whereas the latter is the ability to overcome the cost of distance for using health care services. Economic access is thought to be the prime important factor in the study of health care delivery. Joseph and Phillips divided accessibility into locational accessibility (physical distance) and effective accessibility (e.g., whether a facility is always available or open, and whether it is socially or financially accessible) [7]. In addition, Andersen’s revised Behavioral Model of Health Services Use divides access into four components: potential access (enabling resources), realized access (actual use of health service), equitable access (occurring when resources meet the needs of the population with various demographic characteristics), and inequitable access (occurring when social structure, health beliefs, and enabling resources determine who gets medical care) [8]. The definitions share a common concept of access as multi-dimensional and emphasize the interaction of geographical, economic, social, and cultural factors in the process of access to health care services.

With regard to residential care, a great deal of literature in the U.K. focused on the policy implications of the development of residential care services and variation in the spatial distribution of RCFs. For example, Phillips et al. studied the spatial concentration of residential homes for the elderly in the U.K. [9]. Smith and Ford analysed the changes in the spatial distribution of public and private RCFs between 1988 and 1993 [10]. Both of the studies focused on the spatial distribution and expansion of residential homes at the national and regional scales. Bartlett and Phillips [11] and Andrews and Phillips [12-14] analysed the impacts of policy changes on the development of residential care in the U.K. during the 1980s and 1990s. In the 1980s, private residential care enjoyed substantial state financed support and expanded rapidly. However, since the implementation of the 1990 National Health Service and Care in the Community Act in 1993, the withdrawal of guaranteed state support and intense market competition had negative impacts on many RCFs, which implies a market failure in the social care sector.

With the development of GIS techniques, researchers have applied spatial analysis models to access to health care services. Even though researchers are aware of the importance of both spatial and nonspatial factors in affecting health care access, the earlier research studied the two types of factors separately. For example, Radke and Mu developed the spatial decomposition method (also called the 2SFCA method) to measure access to social services for each household which can help adjust and better accommodate underserved regions [15]. Luo and Wang modified the 2SFCA method developed by Radke and Mu and the gravity-based method to assess the variations in spatial accessibility to primary care in the Chicago region, U.S. [16]. Comparing the two methods, Luo and Wang recommended the 2SFCA method as simpler and more practical for planning and policy making. Later, based on the earlier studies, Wang and Luo took into account geographical and socio-demographic factors [17]. They used an integrated approach to measure accessibility to primary healthcare in Illinois, U.S., which included the 2SFCA method to measure spatial accessibility and factor analysis to measure various socio-demographic factors.

The 2SFCA method, however, has been criticized for its limitations and many improvements have been proposed [18]. For example, Luo and Qi developed an enhanced 2SFCA method (E2SFCA) to address the problem of uniform access within the catchment by applying weights to different travel time zones to account for distance decay [19]. Based on E2SFCA, Luo and Whippo proposed the Variable 2SFCA method (V2SFCA), which adds two more steps to determine the catchment sizes by increasing the catchment until a base population and a physician-to-population ratio met with predefined thresholds [20]. They also used sensitivity analysis to test various thresholds and find out the differences in urban centers and rural areas. The improved method addressed the issue of fixed catchment size and offers a way of determining the proper catchment sizes for both physician and population locations.

The shortest path approach [21], the gravity-based method [22-24] and the 2SFCA method [25] have been most frequently applied to analyse accessibility to health care services in various regions of China. Yin and Xu considered factors such as gender, age and income in a study of accessibility and equity to parks in Shanghai [26]. Cheng conducted a study of residential care in China [27]. She showed that access to residential care is an interconnected process affected by geographical, economic and socio-cultural factors. With regards to geographical access, the study drew a qualitative conclusion that the location of a RCF, convenience of public transportation, the time-distance for families’ and friends’ visits, and access to health care play important roles in choosing a specific RCF. Quantitative methods have been used to analyze health care services, but there is lack of quantitative studies on residential care. More studies using GIS-based methods to analyse accessibility to residential care are urgently needed to contribute to knowledge for future planning and policy making.

### Data and methods

**Figure 1:**
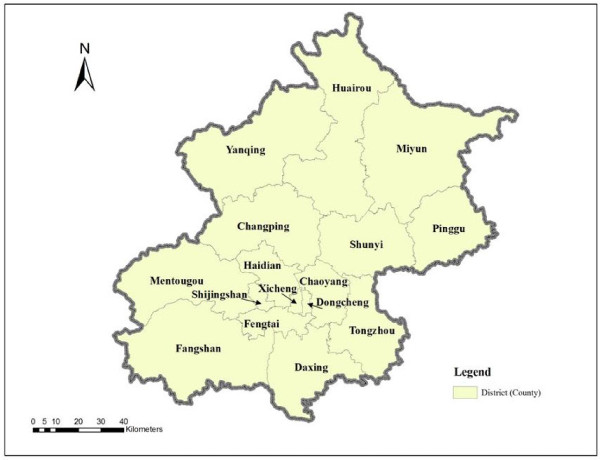
The 14 districts and two counties of Beijing.

Beijing is now divided into 14 districts and two counties (Miyun and Yanqing) after the four districts (Dongcheng, Xicheng, Chongwen, and Xuanwu) in the central city were merged into two districts (Dongcheng and Xicheng) in 2010 (Figure 1). Part of the districts and counties of Beijing, especially some areas in the outskirts are rural in nature and the older people living in those areas are treated as rural residents. The population data at the subdistrict level are from the 2010 National Population Census. As the majority of residential care service users are from the older population, the older population data are calculated based on the total population by multiplying the percentage of the older population by the total population in each district (county). RCF data (e.g., RCF addresses and bed numbers) are from the information provided by the Beijing Municipal Civil Affair Bureau on its website [28]. The latest data are from 2009. Subdistrict (township)^1^ is the smallest areal unit used for the population analysis. RCFs are geocoded based on their street addresses. The data include 307 subdistricts (township) and 332 RCFs in total. Spatial data of the streets and road networks are from National Geomatics Database. 

Two GIS-based methods are used to study the distribution pattern of RCF service areas and spatial accessibility. The first method is based on shortest path analysis. First, the study area is divided into a grid with cells of 10m by 10m, assuming the population has equal access to residential care resources within each cell. According to the road network and class of road, the standard speed is set at 60km/h for expressways, 50km/h for trunk roads, 40km/h for sub-distributors, 30km/h for other roads, and 5km/h within each cell. Second, we calculated the time cost of the shortest path from each cell to the closest target (RCF) based on standard speeds. This method is used to measure the travel time to the closest RCF and the service area of each RCF based on the shortest time distance. Though the shortest path method has been criticized for only considering proximity between population and service location with no account taken of availability, in this study we use the shortest path method to identify residential care shortage areas beyond certain time thresholds, which is important for decision making when one is considering relocation to RCFs [29,30].

The second method is the 2SFCA method first developed by Radke and Mu and later improved upon by Wang and Luo [15,17]. First, for each RCF location *j*, all population locations (*k*) that are within a threshold distance (*d*_*0*_) from location *j* are searched, and the area that is within the distance (*d*_*0*_) from a RCF is drawn as its catchment area, then the bed number of RCFs to population ratio within catchment j, *R*_*j*_, is computed (Formula 1):

(1) $$R_{j}=\frac{S_{j}}{\sum_{k\in \{d_{\mathrm{kj}}\leq d_{o}\}}D_{k}}$$

where *d*_*kj*_ is the distance between *k* and *j*, *D*_*k*_ is the number of older people (demand) of subdistrict (township) *k* whose centroid falls within the catchment (i.e., *d*_*kj*_ *≤ d*_*0*_), and *S*_*j*_ is the number of beds of the RCF at location *j*.

Next, for each population location *i*, we searched all RCF locations (*j*) (supply) that are within the threshold distance (*d*_*0*_) from location *i* (i.e., catchment area *i*), and sum up the number of beds to population ratio *R*_*j*_ at these locations as accessibility $A_{i}^{F}$ (Formula 2):

(2) $$A_{i}^{F}=\sum_{j\in \{d_{\mathrm{ij}}\leq d_{0}\}}R_{j}=\sum_{j\in \{d_{\mathrm{ij}}\leq d_{0}\}}(\frac{S_{j}}{\sum_{k\in \{d_{\mathrm{kj}}\leq d_{0}\}}D_{k}})$$

where $A_{i}^{F}$ represents the accessibility at population location *i* based on the 2SFCA method, *d*_*ij*_ is the distance between *i* and *j*, and *R*_*j*_ is the number of beds to population ratio at RCF location *j* whose centroid falls within the catchment area centered at *i* (i.e., *d*_*ij*_ *≤ d*_*0*_) where the larger a value of $A_{i}^{F}$indicates better accessibility to residential care resources at a population location.

This study is unable to calculate population-weighted centroids of each subdistrict because population data at a smaller scale than the subdistrict (township) level are not available. Therefore, simple geographic centriods of each subdistrict are used instead of population-weighted centroids in the spatial analysis. To simplify the calculation, the distance between any RCF and population location is measured by the straight-line distance instead of time distance. The average distance between any population location and its closest RCF is used as a reference for a reasonable threshold distance (*d*_*0*_ = 3.3km). Straight line distance is frequently used as a proxy measure for travel times and there are studies showing that the observed correlations of travel time and straight-line distance are quite high[31,32]. The first step of the 2SFCA method is to measure the availability of residential care resources for the supply location (RCF), and the second step sums up the initial ratios in the overlapped service areas to measure spatial accessibility for a demand location (population location), where residents have access to multiple RCF locations. The 2SFCA method overcomes some of the limitations of the shortest path analysis. Instead of only taking into account the RCF within the shortest time distance of any population location, the 2SFCA method considers the potential options of RCFs within a distance threshold, as well as the size of the RCFs.

The two spatial analysis methods used in this study are implemented in ArcGIS 9.3 software. “Reclass”, “Raster Calculator”, “Cost Distance”, “Cost Allocation” functions in “Analysis Tools” of ArcGIS9.3 are used for shortest path analysis, and “Proximity”, “Point Distance Tool” functions are used for 2SFCA analysis.

## Results

**Figure 2:**
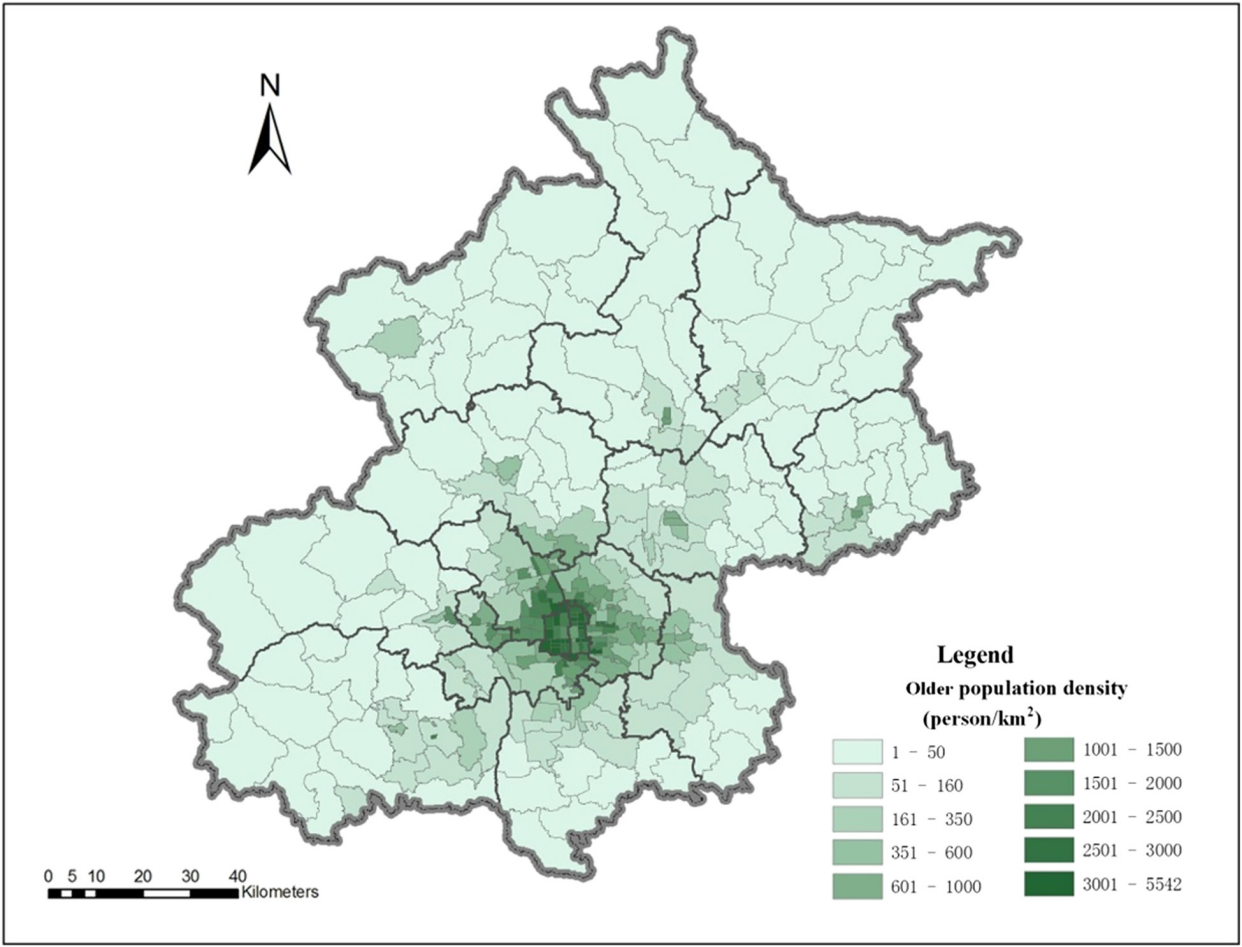
The spatial distribution of older population density at subdistrict level in Beijing in 2010.

The spatial distribution of the older population better reveals the distribution pattern of residential care demand than that of total population, as the majority of service users of residential care are older population. As Figure 2 shows, the older population density is relatively high in the central city and it shows a declining pattern of the older population from the city centre outwards. The highest older population density is 5542.17 older persons per km^2^ in a subdistrict of Dongcheng District at the city centre, while the lowest older population density is only 1.42 older persons per km^2^ in a township of Huairou District. Generally speaking, the older population density in the city centre is higher than 1000 older persons per km^2^, and is lower than 50 older persons per km^2^ in the majority of suburban and exurban areas. The huge variation in the older population density is a strong prima facie argument for the reasonable allocation of care resources.

**Figure 3:**
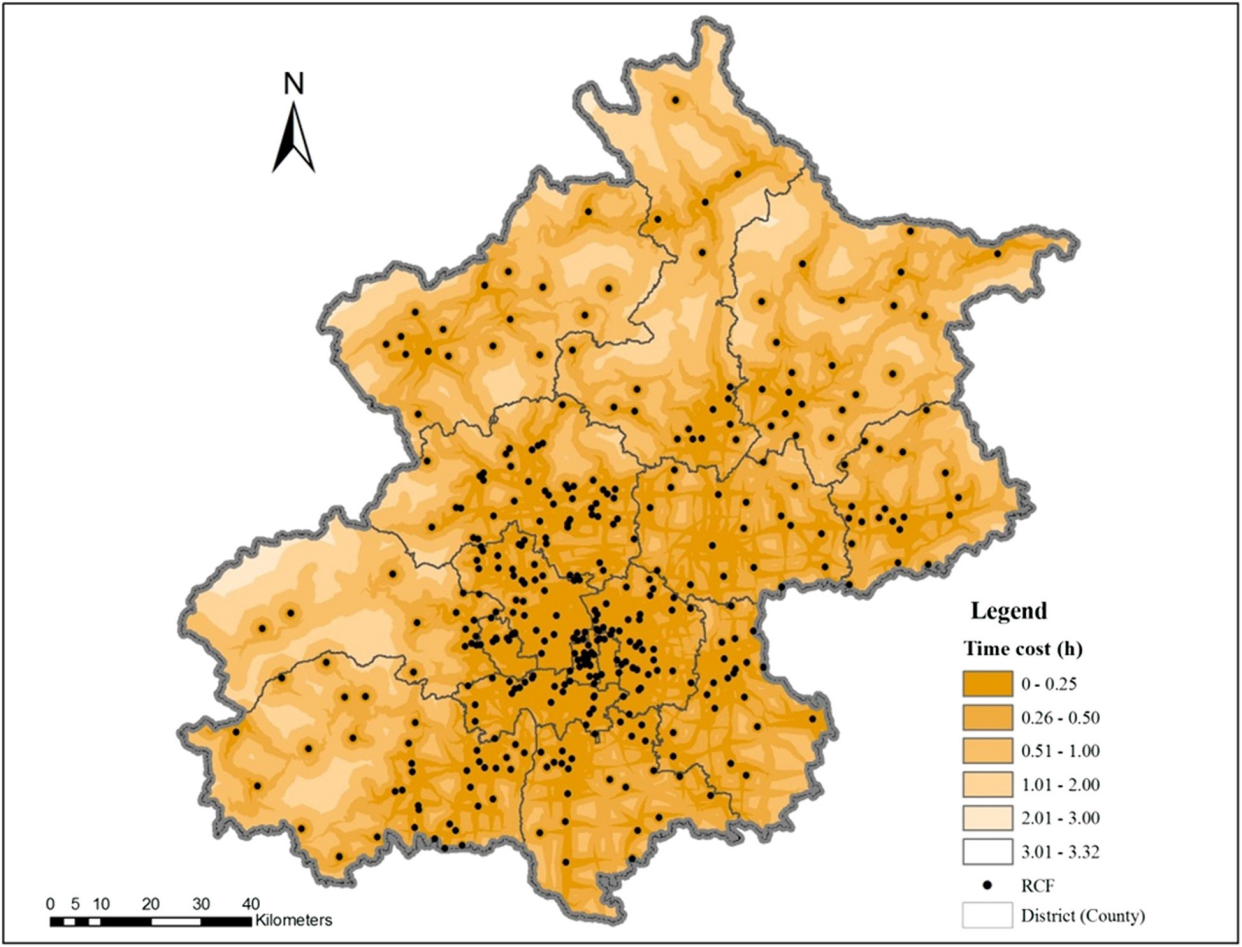
The spatial distribution of travel time to the nearest RCF in Beijing.

The shortest path analysis is used to map the travel time to the nearest RCF at various thresholds (Figure 3). The time distance between population locations and RCFs is highly related to the road network and standard speeds assumed. If only time distance is considered, accessibility to RCFs is better in central districts than in suburban and exurban areas. The time distance from the population locations to their closest RCFs is less than 30 minutes in most areas of the central districts. In the majority of the suburban and exurban areas, the time distance is between 30 minutes and two hours. The spatial accessibility to RCFs is better in the northeast and southeast areas of Beijing than in the northwest and southwest areas.

**Figure 4:**
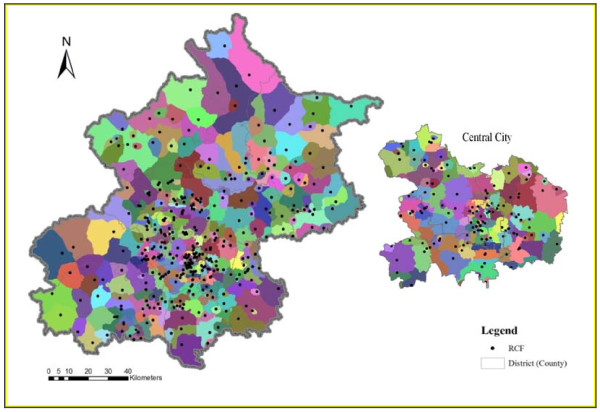
**The spatial distribution of RCF service areas in Beijing.** Note: Each color in the figure represents a different service area.

The results from the shortest path analysis are also used to divide the service area of each RCF which is presented in Figure 4. In the areas with a relatively high density of RCFs, the service area of each RCF is relatively small. In other words, when only time distance is considered, spatial accessibility to residential care resources is relatively high in the areas with a high density of RCFs. As Figure 4 shows, the service areas of RCFs in the central city are relatively small compared to the service areas of RCFs on the outskirts of Beijing, especially in the north and west parts of Beijing.

**Figure 5:**
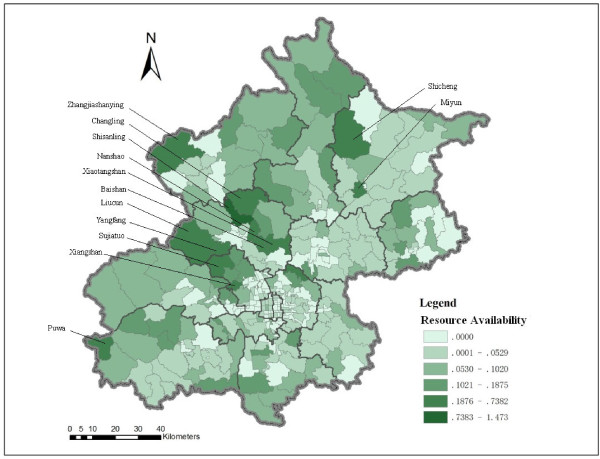
RCF bed number per older person at the subdistrict (township) level in Beijing.

The spatial distribution of available residential care resources in each subdistrict (township) is mapped by using the indicator “bed number per older person” (Figure 5). The spatial distribution of residential care resources is uneven. Different from time distance accessibility, care resources per older person is relatively limited in the central districts, whereas it is relatively abundant in the north and west outskirts of Beijing, especially in the areas near some natural resorts such as the Miyun reservoir (e.g., Shicheng, Miyun), the Ming-tomb reservoir (e.g., Changling, Shisanling, Nanshao, Baishan, Xiaotangshan), the Guanting reservoir (e.g., Zhangshanying), the Fenghuangling area (e.g., Liucun, Yangfang, Sujiatuo), the Xiangshan area (Xiangshan), and Puwa village. The limitation of this indicator is that it assumes older people only access residential care resources in the subdistrict (township) where they live. In reality, older people do not necessarily utilize the care resources offered by the RCF nearest to their homes. They may choose a RCF from several RCFs nearby, or even a RCF in other neighbourhoods considering various factors other than proximity [27]. 

**Figure 6:**
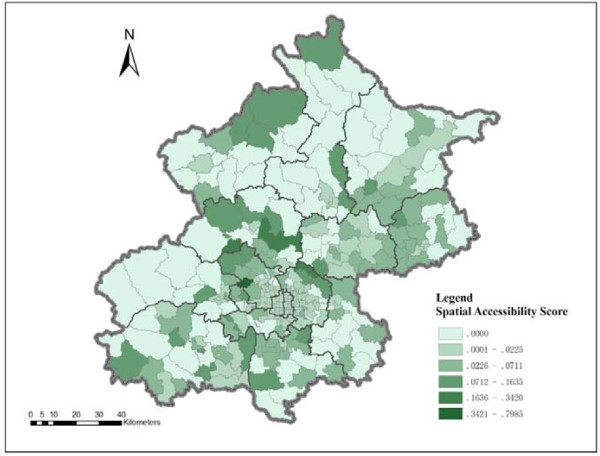
Spatial accessibility to RCF in Beijing by 2SFCA method.

The results show significant variation in spatial accessibility to residential care resources on the left and right sides of a northeast-southwest line (Figure 6). The residential care resources on the right side of the line are generally more accessible than those of the left side. The distribution of residential care resources is more geographically uneven on the left side than the right side of the northeast-southwest division. The residential care resources are generally more accessible in the central city than the majority of outskirts on the left side of the northeast-southwest division. However, several hotspots of RCFs are located in the left side areas, such as the Miyun reservoir, the Ming-tomb reservoir, and Xiangshan area, which can be clearly indentified in Figure 6.

## Discussion

This study analyses the spatial accessibility of RCFs based on population and residential care resources using shortest path analysis and the 2SFCA method. The results from the two methods show different distribution patterns of spatial accessibility to RCFs. Considering time distance, the results from the shortest path analysis show that the spatial accessibility to RCFs is better in the central city (the majority of areas are within 30 minutes distance to the closest RCF) than the outskirts of Beijing (the majority of areas are within two hours distance from the closest RCF). Spatial accessibility decreases as distance from main streets increases. Residential care resources are more spatially accessible in the northeast and southeast areas of the city than the northwest and southwest areas. With regard to RCF care resources, the number of beds per older person is higher in suburban and exurban areas than the central city, and resources in the northwest and southwest areas are more abundant than in the northeast and southeast areas of the city. The 2SFCA method considers geographical distance, older population and number of beds of RCFs. The results show that spatial accessibility to RCFs is relatively poor in the central city, but when compared to the resources in the northwest and west outskirts of the city, the RCF resources in the central city are more spatially accessible. The spatial accessibility to residential care resources in the northeast to southeast side of the city is generally better than that in the northwest to southwest areas, but several hotspots of residential care resources are located in the northwest to southwest outskirts.

The results from both methods are helpful for knowledge-based policy making. Shortest path analysis is able to identify the service areas at certain time thresholds, as well as underserved areas. The qualitative study conducted by Cheng in Beijing found that older people and their family member prefer choosing a RCF within the time distance of one to one and a half hours from their homes [27]. The underserved areas at a certain time threshold (e.g., one and a half hours) are potential areas for allocating more resources. In addition to identifying underserved areas, the 2SFCA method helps provide suggestions for creating a city – district (county) – subdistrict (township) multi-level RCF system based on variation in spatial accessibility to RCFs. For example, the RCFs located in the hotspots with high spatial accessibility scores and those with a large number of beds might be classified as city-level RCFs^2^.

With regard to the data sources, the difference between the two methods is that shortest path analysis is based on raster data and the 2SFCA method is based on vector data in this study. The shortest path analysis only considers the factor of distance. The 2SFCA method considers interaction between demand and supply based on distance, and it calculates the accessibility across administrative border (subdistrict/township border in this case), which reflects service utilization behaviour in real world.

The gravity model is another widely used method to measure accessibility, and it considers a nearby supplier more accessible than a remote one and discounts a supplier’s availability by a gravity-based potential measure [16]. The 2SFCA method is a special case of the gravity-based method. The difference in the two is that distance impedance is dichotomous in the 2SFCA method but continuous in the gravity-based method. For government planning purposes, Luo and Wang suggest that the 2SFCA method is a better choice because the travel friction coefficient in a gravity model varies as the study area changes and defining the coefficient makes the method more complicated [16].

Several limitations of this study should be addressed. First, the quality of geographic information is essential for spatial analysis. In this study, population data are not available at a smaller scale than the subdistrict level. If better quality data were available, then population-weighted centroids instead of simple geographic centroids to represent population locations more accurately would be possible. Second, to simplify the analysis, straight-line distance instead of time distance is used in the 2SFCA method, but existing studies have shown the correlation of straight-line distance and time distance is high. Third, the results need to be interpreted with caution because in this study an arbitrary distance threshold was set in the 2SFCA method, but in reality, older people may utilize residential care resources at a greater distance than the distance threshold. A sensitivity analysis of the distance threshold was not conducted. Existing research shows that the distance threshold/catchment is a critical parameter as it determines the smoothness of the result [16,33-35]. The results of a sensitivity analysis conducted by Luo and Wang show that a larger travel-time threshold generates stronger spatial smoothing and reduces variability of accessibility across space using the 2SFCA method [16]. The smoothing effect of a large catchment size shows that the population to physician ratio of a particular census tract eventually converges to one value as the catchment increases [34]. In a recent study, Shi et al. suggest that different functional forms for determining distance decay weights in the second step of 2SFCA do not significantly impact the spatial pattern of the estimation results [35]. Fourth, the decision making process of relocation is affected by various factors in addition to geographical factors [27]. It is, however, very challenging to examine various factors in the model, although the study conducted by Wang and Luo provides an example of considering both spatial and nonspatial factors in analysing accessibility to primary health care [17]. Access to residential care resources is a complicated process and different from access to other types of health care services because decisions are often not made only by the user (i.e., the older person) but also by other family members and/or friends. Their needs must be taken into account as well.

## Conclusions

In summary from this study, three conclusions can be drawn. First, in terms of time distance, the distribution of RCF service areas is highly related to the road network. The central city has better spatial accessibility than the suburban and exurban areas. The RCF resources in the northeast and southeast areas are more spatially accessible than those in the northwest and southwest areas. In terms of residential care resources, the distribution pattern is opposite of the time distance accessibility pattern, suburban and exurban areas have relatively more abundant resources than the central city. Northwest and southwest areas have more resources per older person than the northeast and southeast areas. Second, considering time distance, older population and RCF resources, the distribution pattern of spatial accessibility to RCFs is more uneven in the northwest and southwest areas than the northeast and southeast areas. The central city has relatively low spatial accessibility, but it is better than the northwest and west outskirts of the city. The results of spatial accessibility vary by using different methods. Whether a method is efficient depends on the research question that one aims to answer. For policy making, it may require combining various methods for a comprehensive analysis. Third, the results provide evidence for future planning of residential care resources, especially a multi-level residential care system. Indeed, with the rapid expansion of residential care services as planned for the next decade, knowledge-based planning is necessary for improving equitable access and the efficient allocation of resources. This study provides valuable information for planning and sheds light on future research by using two GIS-based spatial analysis methods.

### Endnotes

1. Currently, the constitution of China provides for five levels of local governments: the province, prefecture (city), county (district), township (subdistrict), and village (neighbourhood). Township is a rural administrative unit under the county and subdistrict is an urban administrative unit under the district.

2. City-level RCFs refer to the RCFs which provide various types of service for older people living in Beijing and they are usually large in size. They are different from district-level RCFs or community-level RCFs for older people living in the district or community where the RCF locates.

## Abbreviations

GIS: Geographic Information System; 2SFCA: Two-step floating catchment area; RCF: Residential care facility.

## Competing interests

All authors declare that they have no competing of interest.

## Authors’ contributions

YC and JEW contributed to the design and analysis of the study and writing of the manuscript. MWR contributed to drafting the manuscript and revising it. All authors read and approved the final manuscript.
